# Simultaneous, Quantitative Detection of Four Common *Vibrio* Species by Microfluidic Chamber-Based Digital PCR in Aquatic Products

**DOI:** 10.3390/foods15142547

**Published:** 2026-07-19

**Authors:** Haibo Zhou, Na Wang, Xinmei Liu, Ning Liu, Xiaomei Bie, Jun Yang

**Affiliations:** 1 Key Laboratory of Detection and Traceability Technology of Foodborne Pathogenic Microorganisms, State Administration for Market Regulation, Key Laboratory of Detection and Traceability Technology of Foodborne Pathogenic Bacteria for Jiangsu Province Market Regulation, Nanjing Institute for Food and Drug Control, Nanjing 211198, China; zhouhaibo163@163.com (H.Z.);; 2 College of Food Science and Technology, Nanjing Agricultural University, Nanjing 210095, China

**Keywords:** *Vibrio* species, chamber-based digital PCR, target genes, multiplex assay, quantitative detection

## Abstract

*Vibrio* spp., known as typical marine pathogens, pose serious threats to human health worldwide due to their high incidence and prevalence. In this study, a quantitative detection method was developed for *Vibrio parahaemolyticus*, *Vibrio vulnificus*, *Vibrio cholerae* and *Vibrio alginolyticus* in aquatic products. A set of multiplex chamber-based digital PCR (cdPCR) primers and probes was designed based on new molecular targets of *Vibrio* species. The conditions of multiplex cdPCR were optimized and compared with those of multiplex qPCR. Under optimal conditions, the performance indicators, including the specificity, linear range, and detection limit, were systematically evaluated. The results indicate that the established method had 100% specificity among all tested strains and did not cross-react with other foodborne pathogens tested in parallel. The linear dynamic range was 10^2^–10^6^ CFU/mL, and the limits of detection (LODs) for *V. parahaemolyticus*, *V. vulnificus*, *V. cholerae* and *V. alginolyticus* were 5.40 × 10^2^ CFU/mL, 3.05 × 10^2^ CFU/mL, 4.55 × 10^2^ CFU/mL and 1.32 × 10^2^ CFU/mL, respectively. Furthermore, the feasibility of the proposed method was assessed in contaminated food samples, with a 10-fold improvement in detection sensitivity over that of the qPCR method under the conditions of this study. The entire detection process could be completed within 2 h. Taken together, the results of this study offer a readily adaptable diagnostic method, allowing for the simultaneous and quantitative detection of four pathogenic *Vibrio* species, which is promising for food safety and environmental monitoring.

## 1. Introduction

Over the past few decades, the rapid development of fisheries and aquaculture has met the growing demand for aquatic animal protein among the global population. Global aquaculture production reached a new high of 130.9 million tonnes in 2022, of which Asia accounted for 91.4% of the overall total; the per capita consumption of aquatic animal foods increased from 9.1 kilograms per year in 1961 to 20.6 kilograms per year in 2021 [1]. *Vibrio* spp. bacteria are naturally distributed in coastal waters and aquatic products (such as fish, shellfish and crustacea, particularly oysters), and the most important species causing human illnesses are *Vibrio parahaemolyticus*, *Vibrio vulnificus*, *Vibrio cholerae* and *Vibrio alginolyticus* [2,3]. Among them, illnesses caused by *V. parahaemolyticus*, *V. vulnificus*, and *V. alginolyticus* are called vibriosis, and 80,000 cases are reported in the United States every year [4]. Illnesses caused by *V. cholerae* are defined as those caused by cholera; an estimated 1.3–4 million people around the world develop cholera, and between 21,000 and 143,000 people die [5]. Importantly, *Vibrio* species can also be frequently detected in aquatic products, posing a great risk to human health and food safety. Thus, the development of a reliable, accurate, sensitive, and quantitative detection method for the simultaneous monitoring of four common *Vibrio* species during food production, processing, storage and transportation is urgently needed.

As seen from the National Food Safety Standard (GB 4789 series) [6,7], chromogenic media and biochemical identification have been widely applied in microbial detection. Although traditional culture methods are relatively mature and widely standardized, they are cumbersome, time-consuming, and labor intensive. In recent years, numerous nucleic acid-based detection methods, including real-time quantitative PCR (qPCR) [8,9], loop-mediated isothermal amplification (LAMP) [10,11,12] and recombinase polymerase amplification (RPA) [13,14], have been developed to meet the timeliness requirements for testing in food safety supervision, as well as among enterprises and industries. Although these methods offer portability, flexibility and speed, they often support qualitative or relatively quantitative detection. Additionally, digital PCR (dPCR) is emerging as a reliable technique for quantifying nucleic acid samples. Unlike real-time monitoring, which is based on signal accumulation, dPCR is an end-point technology that changes the standard from fluorescence intensity to the presence or absence of a detected fluorescent signal. Specifically, a single original DNA molecule is individually amplified after the reaction system is partitioned into thousands or millions of units, and absolute quantification is achieved using Poisson statistics by counting the numbers of positive and negative chambers [15,16]. On the other hand, the dynamic range of dPCR (typically 4 to 5 orders of magnitude) is constrained by the gene copy number range spanning from zero positive droplets to 100% positive droplets, which is the core feature of dPCR and directly affects the accuracy of the results. Therefore, dPCR offers improved capabilities, including better tolerance, superior accuracy, higher sensitivity, and reduced dependency on amplification efficiency. As a new generation of nucleic acid quantification technology, dPCR has been increasingly applied in genetically modified organism (GMO) analysis [17,18], cancer diagnosis [19,20], and microbiology detection [21,22,23,24,25]. In terms of the partition type, dPCR can be classified into two main types: droplet-based digital PCR (ddPCR) and chamber-based digital PCR (cdPCR). The compartments of ddPCR are nanoliter-sized droplets in the form of a water-in-oil emulsion using a specialized droplet generator, and reading out of the droplets requires a specialized microfluidic reader. Compared with ddPCR, cdPCR, which features predefined well locations, is considered to have greater uniformity and lower contamination risks due to the integration of multiple steps, such as reaction partitioning, amplification, and signal detection [16]. Currently, most reported dPCR assays are developed on the ddPCR platform, and capable of identifying 1 to 3 targets in a single reaction [21,24,26].

In general, the commonly used nucleic acid detection targets for four *Vibrio* species include outer membrane protein genes, virulence genes and DNA gyrase genes. However, several shortcomings have been identified in practical applications, necessitating the exploration of novel targets. In this study, a quantitative method capable of simultaneously detecting four *Vibrio* species was innovatively established using microfluidic cdPCR. To achieve this goal, the self-screened genes *vpa1585*, *vv08030*, *vc09280* and *va01740* were targeted specifically to detect *V. parahaemolyticus*, *V. vulnificus*, *V. cholerae,* and *V. alginolyticus*, respectively. The reaction system and amplification procedure were optimized to enhance the performance and cost-effectiveness of 4-plex cdPCR, and its specificity, sensitivity and applicability were thoroughly evaluated. The most effective combination of established methods was applied to the comprehensive surveillance of pathogenic *Vibrio* species in aquatic products and provides potential application prospects in multiple fields.

## 2. Materials and Methods

### 2.1. Bacterial Strains and Reagents

All the strains used in this study were stored in the Microbiology Laboratory at the Nanjing Institute for Food and Drug Control and are shown in Table 1. Among them, 32 *Vibrio* isolates were isolated from aquatic products and confirmed by both matrix-assisted laser desorption/ionization time-of-flight mass spectrometry (MALDI-TOF MS; Bruker Daltonic, Billerica, MA, USA) and a VITEK-2 microbiology analysis system (BioMérieux, Marcy I’Etoile, France). Sixteen standard strains were obtained from the American Type Culture Collection (ATCC), China Center of Industrial Culture Collection (CICC), and China Medical Culture Collection (CMCC). Absolute Q™ DNA Digital PCR Master Mix (5×) and QuantStudio™ Absolute Q™ Isolation Buffer were purchased from Applied Biosystems (Foster City, CA, USA). Premix Ex Taq (Probe qPCR) was purchased from Takara (Dalian, China). Three percent (*w*/*v*) NaCl alkaline peptone water (APW), thiosulfate-citrate-bile salts–sucrose (TCBS) agar and lysogeny broth (LB) were purchased from Beijing Land Bridge (Beijing, China). *Vibrio* chromogenic agar was purchased from HuanKai Microbial (Guangzhou, China).

### 2.2. Target Genes, Primers and Probes

The target genes of *V. parahaemolyticus*, *V. vulnificus* and *V. alginolyticus* were screened using bioinformatics approaches, which demonstrated good specificity in previous studies [27]. The *vpa1585* gene (*V. parahaemolyticus*; GenBank: BA000032.2; region: 1681983–1682471), *vv08030* gene (*V. vulnificus*; GenBank: CP012881.1; region: 205827–206744), and *va01740* gene (*V. alginolyticus*; GenBank: CP006718.1; region: 399130–400662) were targeted. Similarly, the self-screened gene *vc09280* (GenBank: CP013317.1; region: 1976713–1977453) was identified as the target gene of *V. cholerae* using the method described in our previous work [28]. Primers and probes were designed based on conserved sequences using Primer Premier 5.0 software and the details are listed in Table 2. The specificity of the primers was analyzed with the Primer-BLAST tool (https://www.ncbi.nlm.nih.gov/tools/primer-blast) (accessed on 9 January 2025) at the National Center for Biotechnology Information (NCBI, USA). All the primers and probes used in this study were synthesized by GenScript Biotech Co., Ltd. (Nanjing, China).

### 2.3. Bacterial Cultivation and DNA Extraction

The *Vibrio* bacterial strains, including *V. parahaemolyticus*, *V. vulnificus*, *V. cholerae* and *V. alginolyticus*, were cultured in 3% NaCl APW medium for 18 h. The other non-*Vibrio* strains were cultured in LB medium for 18 h. Genomic DNA extraction was performed using the heat lysis method. One milliliter of fresh culture was centrifuged for 5 min at 6000× *g*, and the supernatant was carefully discarded. The bacterial precipitate was resuspended thoroughly in 100 μL of distilled sterilized water. The tube was boiled in a metal bath at 100 °C for 10 min and then chilled in an ice bath for 10 min. After centrifugation at 14,000× *g* for 5 min, the clear colorless supernatant containing the extracted DNA was transferred into a new centrifuge tube, which was used as the target template for cdPCR and qPCR detection.

### 2.4. Multiplex cdPCR and Multiplex qPCR

Multiplex cdPCR was performed in a final volume of 10 μL, which included 1×Absolute Q™ DNA Digital PCR Master Mix; 0.40 μM of *vpa1585*, *vv08030*, *vc09280* and *va01740* primers; 0.05 μM of *vpa1585* probe; 0.30 μM of *vv08030* probe; 0.25 μM of *vc09280* probe; 0.50 μM of *va01740* probe; 0.25 μL of DNA template, and the remaining volume was filled with sterile distilled water. To generate the droplets, 9 μL of this reaction mixture was added to the wells of the MAP16 plate, and 15 μL of isolation buffer was then overlaid on the reaction mixture, which was immediately covered with a seal (Applied Biosystems) according to the manufacturer’s instructions. The thermal cycling program was as follows: predenaturation at 96 °C for 10 min, followed by 40 cycles of denaturation at 96 °C for 15 s and annealing-extension at 59 °C for 30 s. After amplification, four-channel signals were analyzed using Absolute Q™ Digital PCR Software (version 6.2.1, Applied Biosystems). *vpa1585* with the FAM signal was monitored in channel 1, *vv08030* with the HEX signal was monitored in channel 2, *vc09280* with the TAMRA signal was monitored in channel 3, and *va01740* with the CY5 signal was monitored in channel 4. Negative droplets with lower fluorescent signals and positive droplets with higher fluorescent signals were effectively distinguished by setting a fluorescence amplitude threshold. Finally, the gene copies were calculated using the following formula: C = C_0_ × V_0_/(V_1_ × V_2_), where C_0_ is the copy number in the 10 μL reaction system (copies/µL), V_0_ is the final volume of extracted DNA (µL), V_1_ is the volume of DNA template in the 10 μL reaction system (µL), and V_2_ is the volume of bacterial solution for DNA extraction (mL).

The multiplex qPCR mixture (25 μL) consisted of 1×Premix Ex Taq (Probe qPCR); 0.16 μM of *vpa1585* and *vv08030* primers; 0.08 μM of *vc09280* primers; 0.24 μM of *va01740* primers; 0.08 μM of *vpa1585* and *vv08030* probes; 0.04 μM of *vc09280* probe; 0.20 μM of *va01740* probe; 0.5 μL of DNA template; and sterile distilled water up to 25 μL. qPCR was carried out in a C1000 Touch™ Thermal Cycler (Bio-Rad, Hercules, CA, USA) under the following program: predenaturation at 95 °C for 30 s, followed by 40 cycles of denaturation at 95 °C for 5 s and annealing-extension at 60 °C for 30 s (fluorescence collection).

### 2.5. Optimization of Multiplex cdPCR

Considering the fluorescence intensity and cost, the concentrations of primers and probes were optimized first under singleplex cdPCR. Based on the results, an orthogonal experiment L_9_(3^4^) with four factors and three levels was adopted to determine the optimal probe concentration of multiplex cdPCR, and nine different combinations are listed in Table S1. The annealing temperature is a key parameter for the thermal cycling program and influences the amplification specificity and efficiency of cdPCR. Five different annealing temperatures (58, 59, 60, 61, and 62 °C) were tested at the optimal concentration of the probes. Thus, distinguishing between positive and negative droplets was regarded as the main evaluation criterion.

### 2.6. Specificity Analysis of Multiplex cdPCR 

Specificity is one of the important parameters for evaluating method performance. *V. parahaemolyticus* ATCC 17802, *V. vulnificus* ATCC 27562, *V. cholerae* CICC 23794, *V. alginolyticus* ATCC 17749 and target bacterial isolates were used as positive controls, whereas five non-*Vibrio* strains and other nontarget strains served as negative controls. Genomic DNA was extracted to assess the specificity of the established method.

### 2.7. Sensitivity Evaluation of Multiplex cdPCR

To evaluate the sensitivity of the multiplex cdPCR method, logarithmic-phase bacterial cultures were used for plate colony counting. The mixture of the four *Vibrio* species at a ratio of 1:1:1:1 was serially diluted to obtain 10^1^ CFU/mL to 10^6^ CFU/mL samples. The DNA templates of the above gradient were extracted by the thermal lysis method and used to evaluate the linear range and sensitivity of the system. For multiplex cdPCR, a standard curve was plotted to determine the direct relationship between the DNA copy number (log_10_ copies/mL) and bacterial concentration (log_10_ CFU/mL). For multiplex qPCR, a standard curve was plotted to determine the direct relationship between the cycle threshold (Ct or Cq) values and bacterial concentration (log_10_ CFU/mL).

### 2.8. Artificial Contamination Sample Detection

The salmon, shrimp, raw oyster and large yellow croaker samples were purchased from a local supermarket (Nanjing, China) and confirmed to be free of *V. parahaemolyticus*, *V. vulnificus*, *V. cholerae* and *V. alginolyticus* using a culture method with both TCBS and *Vibrio* chromogenic agar. A total of 25 g of sample was mixed with 2.5 mL of bacterial suspension at varying concentrations (10^3^–10^8^ CFU/mL). Then, 225 mL of 3% NaCl APW medium was added and fully homogenized using a BagMixer (Interscience, Saint Nom, France) for 3 min. One milliliter of artificially contaminated sample was collected for detection using the proposed multiplex cdPCR method.

### 2.9. Statistical Analysis

The Cq values from qPCR were automatically generated using CFX Maestro software (version 4.1.2433.1219, Bio-Rad), and the results were considered positive when the Cq value of each target was ≤36 [21]. The copy number of the cdPCR was obtained using QuantStudio™ Absolute Q™ Digital PCR software (version 6.2.1, Applied Biosystems) and the result was considered valid when the relative standard deviation (RSD) of each target was ≤25% [29,30]. Statistical analysis was performed with 95% confidence intervals using GraphPad Prism software (version 8.0.1, GraphPad Software Inc., USA). Cohen’s kappa values were calculated using the GraphPad online calculator (https://www.graphpad.com/quickcalcs/kappa1) (accessed on 4 July 2026). All experiments were conducted in triplicate.

## 3. Results

### 3.1. Optimization of the Primer/Probe Ratio in Singleplex cdPCR

**Figure 1:**
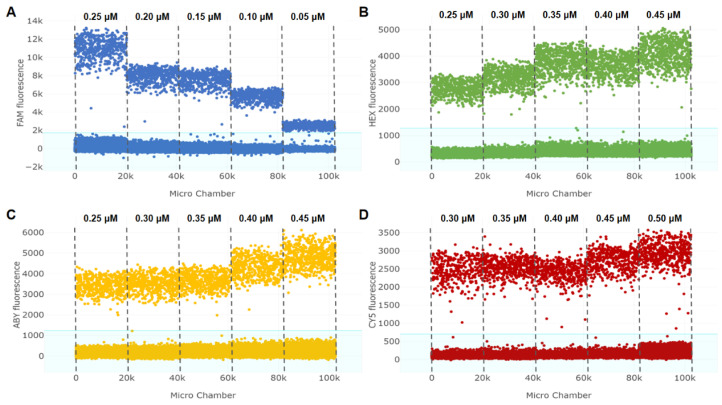
Optimization of the probe concentration in the singleplex cdPCR system. (**A**) *vpa1585* (FAM). (**B**) *vv08030* (HEX). (**C**) *vc09280* (TAMRA). (**D**) *va01740* (CY5). Droplet colors indicate which target was amplified: *vpa1585* (blue), *vv08030* (green), *vc09280* (yellow), and *va01740* (red). The threshold is shown as a light blue line (same as below).

To establish the optimal multiplex cdPCR system for detecting *V. parahaemolyticus*, *V. vulnificus*, *V. cholerae* and *V. alginolyticus*, the primer/probe ratio was initially optimized for singleplex cdPCR to detect each individual target. As shown in Figure 1, the fluorescence value of positive droplets tended to increase with decreasing primer/probe ratio (increase in probe). In addition, the amplification efficiency clearly varied for different targets. Considering that an excessive number of probes does not improve the resolution, we adopted a fluorescence intensity of 2000–4000 as the screening criterion. Hence, the probe concentrations for *V. parahaemolyticus*, *V. vulnificus*, *V. cholerae* and *V. alginolyticus* were determined to be 0.05, 0.30, 0.25, and 0.50 μM, respectively.

**Figure 2:**
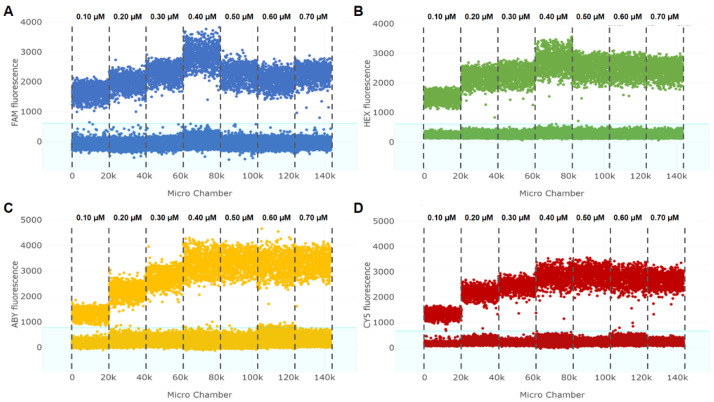
Optimization of the primer concentration in the singleplex cdPCR system. (**A**) *vpa1585* (FAM). (**B**) *vv08030* (HEX). (**C**) *vc09280* (TAMRA). (**D**) *va01740* (CY5).

An appropriate amount of primer can guarantee the sufficient cleavage of fluorescent probes. Seven different concentrations of primer (0.10, 0.20, 0.30, 0.40, 0.50, 0.60, and 0.70 μM) were tested at the optimal probe dosage. As depicted in Figure 2, the fluorescence intensity of positive droplets increased with increasing primer concentration within a certain range, but saturation occurred when the primer concentration exceeded 0.40 μM. With primer concentrations of 0.50, 0.60, and 0.70 μM, no significant changes in the fluorescence value were detected, which indicated the close relationships among the generation of amplification products, the efficiency of probe binding and hydrolysis, and the intensity of fluorescent signals. The optimal primer/probe concentration ratios of *V. parahaemolyticus*, *V. vulnificus*, *V. cholerae* and *V. alginolyticus* were determined to be 8:1, 4:3, 8:5, and 4:5, respectively.

### 3.2. Determination of the Probe Concentrations in Multiplex cdPCR

**Figure 3:**
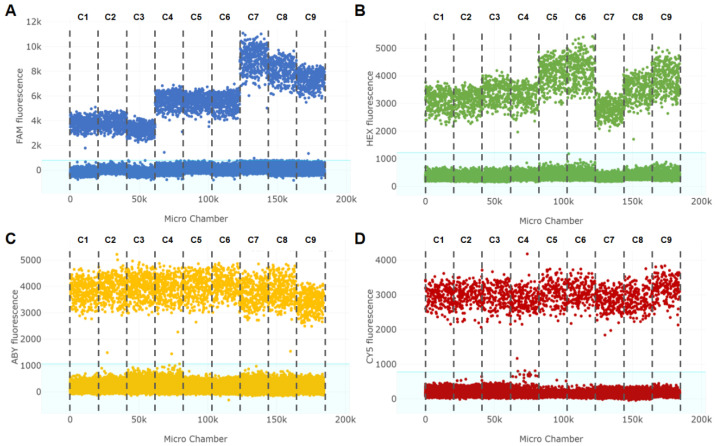
Optimization of probe dosage in the 4-plex cdPCR. (**A**) *vpa1585* (FAM). (**B**) *vv08030* (HEX). (**C**) *vc09280* (TAMRA). (**D**) *va01740* (CY5). C1–C9: different combinations in the orthogonal experiment L_9_(3^4^).

Further experimental studies to explore the combination of four *Vibrio* species were warranted. In accordance with the results of the single-factor experiments, the probe concentrations for *V. parahaemolyticus* (0.05, 0.10, 0.15 μM), *V. vulnificus* (0.30, 0.35, 0.40 μM), *V. cholerae* (0.25, 0.30, 0.35 μM) and *V. alginolyticus* (0.50, 0.55, 0.60 μM) were optimized in the same reaction. Figure 3 displays the different combinations of *vpa1585* (Figure 3A), *vv08030* (Figure 3B), *vc09280* (Figure 3C) and *va01740* (Figure 3D) probes. For the *vpa1585* gene, the fluorescence intensity increased gradually as the probe dosage increased, and a similar phenomenon occurred for the *vv08030* gene. Interestingly, no obvious change in fluorescence signal was observed for the *vc09280* and *va01740* genes. After comprehensive consideration of the resolution and reagent cost, combination 1 with comparable fluorescence intensity (3000–4000) was regarded as the optimal combination of the four *Vibrio* species in multiplex cdPCR.

### 3.3. Optimization of the Annealing Temperature in Multiplex cdPCR

**Figure 4:**
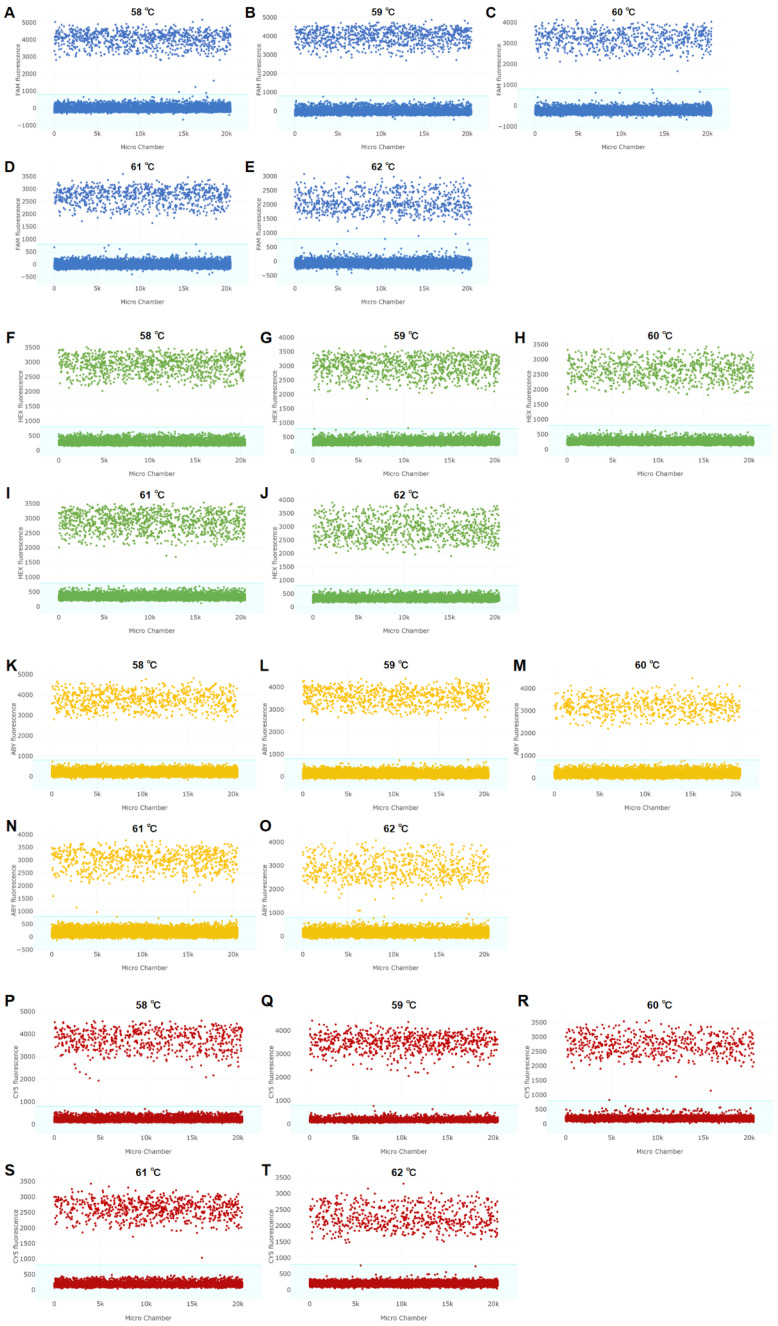
Optimization of the annealing temperature in the 4-plex cdPCR. (**A**–**E**) *vpa1585* (FAM). (**F**–**J**) *vv08030* (HEX). (**K**–**O**) *vc09280* (TAMRA). (**P**–**T**) *va01740* (CY5).

Annealing temperature is critical for the specificity, efficiency and clustering quality of cdPCR, especially for multiple detection systems. Specifically, too low of an annealing temperature increases the tolerance for primer and probe quality but may cause nonspecific amplification, and too high of an annealing temperature inhibits nonspecific amplification but may reduce amplification efficiency. Therefore, it is necessary to select an appropriate annealing temperature to balance the compatibility of multitarget reactions. Overall, except for the HEX channel (*vv08030*, Figure 4B), the other channels (Figure 4A,C,D) exhibited a decrease in fluorescence intensity as the annealing temperature increased, indicating that the *vv08030* primer/probe set has better temperature tolerance. When the annealing temperature was 58 °C, the fluorescence signal value of the system was the highest. A temperature of 59 °C was determined as the optimal annealing temperature for the reaction after consideration of the specificity and comparable fluorescence intensity.

### 3.4. Specificity of Multiplex cdPCR

**Figure 5:**
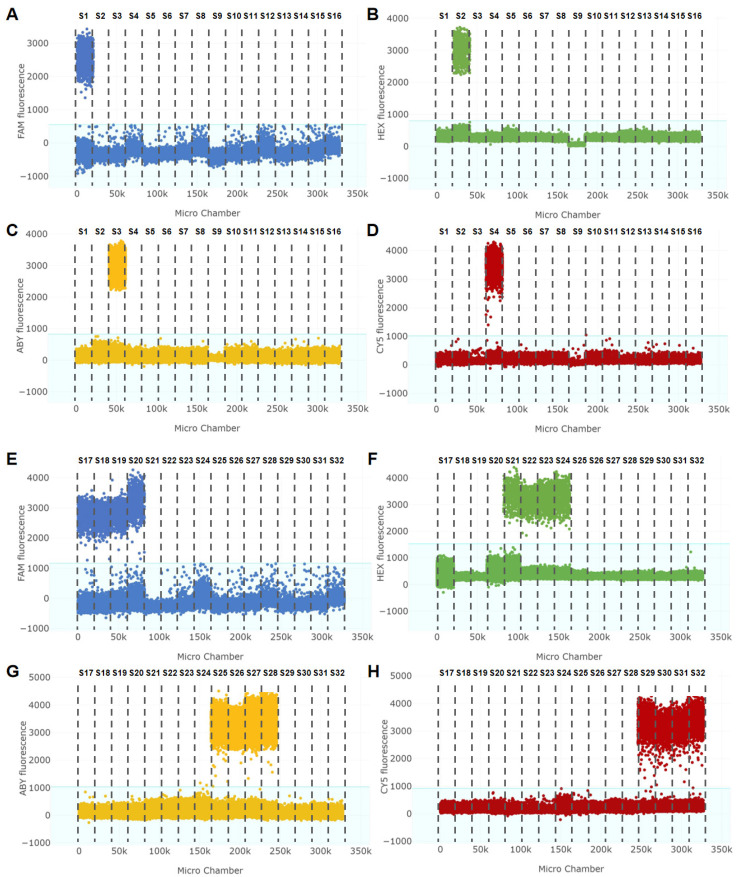

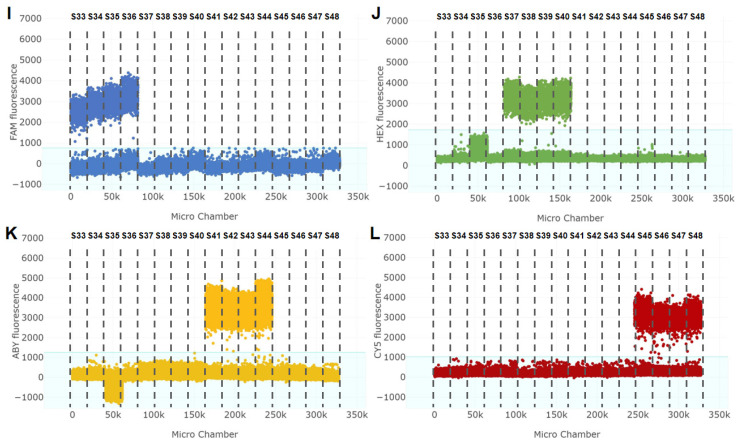
Specificity test results of the 4-plex cdPCR assay. (**A**,**E**,**I**) *vpa1585* (FAM). (**B**,**F**,**J**) *vv08030* (HEX). (**C**,**G**,**K**) *vc09280* (TAMRA). (**D**,**H**,**L**) *va01740* (CY5). S1–S16: ATCC 17802, ATCC 27562, CICC 23794, ATCC 17749, ATCC 33564, ATCC 33842, ATCC 33809, ATCC 33653, ATCC 700040, ATCC 14028, ATCC 25923, ATCC 19115, ATCC 9027, ATCC 25922, ATCC 29544, CMCC(B) 51572. S17–S32: Vp1, Vp2, Vp3, Vp4, Vv1, Vv2, Vv3, Vv4, Vc1, Vc2, Vc3, Vc4, Va1, Va2, Va3, Va4. S33–S48: Vp5, Vp6, Vp7, Vp8, Vv5, Vv6, Vv7, Vv8, Vc5, Vc6, Vc7, Vc9, Va5, Va6, Va7, Va8.

Specificity is the primary parameter for evaluating the performance of a method. In this study, the specificity of multiplex cdPCR was evaluated using the genomic DNA of 36 target bacterial strains and 12 nontarget bacterial strains as templates. As displayed in Figure 5, the four standard strains of *Vibrio* species (ATCC 17802, ATCC 27562, CICC 23794 and ATCC 17749) and 32 isolates were positive for droplets, indicating that the multiplex cdPCR assay was specific for the detection of *V. parahaemolyticus*, *V. vulnificus*, *V. cholerae* and *V. alginolyticus*. The five negative strains of *Vibrio* spp. (*V. metschnikovii*, *V. mimicus*, *V. fluvialis*, *V. hollisae* and *V. harveyi*) and seven other strains of common foodborne pathogens showed only negative droplets, indicating that the assay did not cause cross-reactions. Therefore, the multiplex cdPCR platform established in this study had satisfactory specificity for the detection of *V. parahaemolyticus*, *V. vulnificus*, *V. cholerae* and *V. alginolyticus*.

### 3.5. Standard Curve and Sensitivity of Multiplex cdPCR

**Figure 6:**
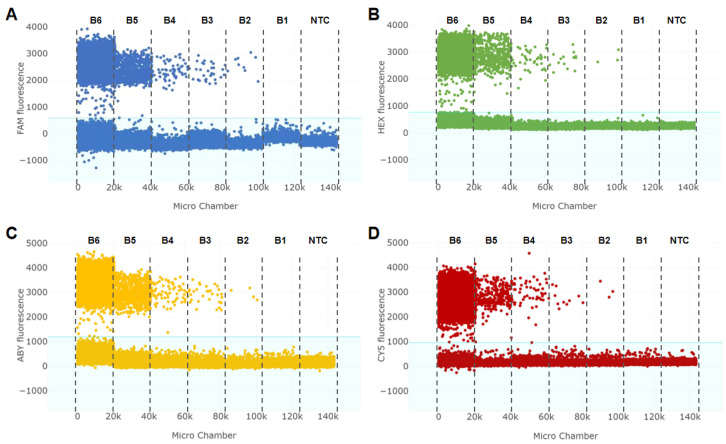
Sensitivity evaluation results of 4-plex cdPCR detection. (**A**) *vpa1585* (FAM). (**B**) *vv08030* (HEX). (**C**) *vc09280* (TAMRA). (**D**) *va01740* (CY5). B1–B6: 10^1^–10^6^ CFU/mL. NTC, no-template control.

To test the sensitivity of our multiplex cdPCR to the bacterial suspension template, six 10-fold serially diluted positive samples from 10^1^ to 10^6^ CFU/mL were prepared. It was easy to distinguish positive and negative droplets, and the copy number of the target DNA template was calculated by Poisson statistics based on the proportion of positive reactions. As shown in Figure 6, the number of positive droplets decreased with the dilution of target templates, whereas the no-template control did not show a positive signal. All target genes exhibited linear performance, and the linear range was from 10^2^–10^6^ CFU/mL. Furthermore, standard curves were established by plotting bacterial concentrations against copy numbers, and the correlation equations were log_10_ copies/mL = 0.9144 log_10_ CFU/mL − 1.8261 (R^2^ = 0.9946) for *vpa1585*, log_10_ copies/mL = 0.9299 log_10_ CFU/mL − 1.8191 (R^2^ = 0.9912) for *vv08030*, log_10_ copies/mL = 1.0246 log_10_ CFU/mL − 2.4011 (R^2^ = 0.9997) for *vc09280*, and log_10_ copies/mL = 0.9646 log_10_ CFU/mL − 2.1702 (R^2^ = 0.9979) for *va01740*. A bacterial concentration of 10^1^ CFU/mL was also evaluated; three replicates of the experiments were carried out, and two of them failed to show positive reactions. Thus, the LODs of *V. parahaemolyticus*, *V. vulnificus*, *V. cholerae* and *V. alginolyticus* were determined to be 5.40 × 10^2^ CFU/mL, 3.05 × 10^2^ CFU/mL, 4.55 × 10^2^ CFU/mL and 1.32 × 10^2^ CFU/mL, respectively. As these levels met the quantitative criterion (RSD ≤ 25%), they were also defined as the limits of quantitation (LOQs) of the method.

**Figure 7:**
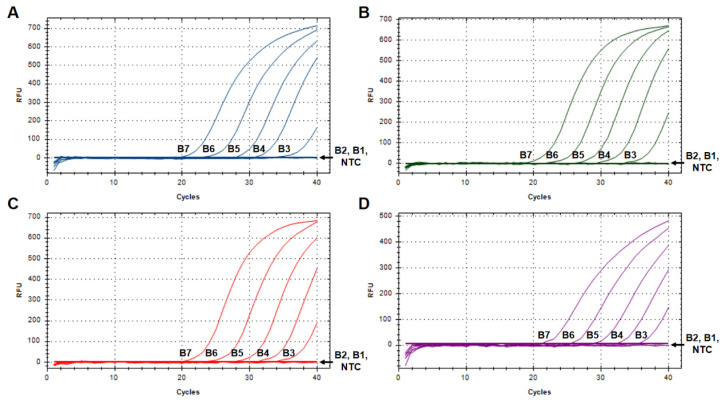
Sensitivity evaluation results of 4-plex qPCR detection. (**A**) *vpa1585* (FAM). (**B**) *vv08030* (HEX). (**C**) *vc09280* (Texas Red). (**D**) *va01740* (CY5). B1–B7: 10^1^–10^7^ CFU/mL. NTC, no-template control.

For multiplex qPCR (Figure 7), standard curves were constructed by plotting bacterial concentrations against Cq values, and the correlation equations were Cq = −3.442 log_10_ CFU/mL + 46.73 (R^2^ = 0.986) for *vpa1585*, Cq = −3.649 log_10_ CFU/mL + 46.74 (R^2^ = 0.995) for *vv08030*, Cq = −3.496 log_10_ CFU/mL + 46.65 (R^2^ = 0.994) for *vc09280*, and Cq = −3.259 log_10_ CFU/mL + 46.40 (R^2^ = 0.991) for *va01740*. The LOD of all target genes reached only 10^3^ CFU/mL, which was 10-fold lower than that of the multiplex cdPCR (kappa = 0.588). Collectively, these results indicated that the multiplex cdPCR method could be utilized for the quantitative detection of four *Vibrio* species with high sensitivity and convenience.

### 3.6. Application of Multiplex cdPCR in Artificially Contaminated Samples

To validate the practicability of the methods established, we detected *V. parahaemolyticus*, *V. vulnificus*, *V. cholerae* and *V. alginolyticus* in artificially contaminated samples. Four standard strains (ATCC 17802, ATCC 27562, CICC 23794 and ATCC 17749) were spiked into actual samples at final concentrations ranging from 10^1^ to 10^6^ CFU/mL, and all detection assays were performed directly without any prior enrichment. The results show that the multiplex cdPCR could reach detection limits as low as 4.36 × 10^2^ CFU/mL for *V. parahaemolyticus*, 2.65 × 10^2^ CFU/mL for *V. vulnificus*, 5.46 × 10^2^ CFU/mL for *V. cholerae* and 3.24 × 10^2^ CFU/mL for *V. alginolyticus*, as shown in Table 3 and Tables S2–S4. The RSDs of positive samples with different contamination levels were all less than 25%, which was within the acceptable range. When multiplex qPCR was used, fluorescence signals were observed only when the bacterial concentration was above 10^3^ CFU/mL. Additionally, the stability of cdPCR detection is superior to that of qPCR when bacteria are targeted at relatively low concentrations (10^3^ CFU/mL), further highlighting the good feasibility of multiplex cdPCR in practical applications.

## 4. Discussion

Over the past decade, many point-of-care testing (POCT) technologies, such as CRISPR/Cas systems [31,32], biosensors [33,34,35], and lateral flow assays [36,37], have been proposed to meet the requirements of onsite detection. However, it is difficult to achieve accurate quantification of single or multiple targets in practice; such methods rely heavily on correlations between signal change and target concentration. Studies have shown that the detection limit of dPCR is one order of magnitude lower than that of qPCR [38,39]. Therefore, we constructed a multiplex cdPCR detection method for *V. parahaemolyticus*, *V. vulnificus*, *V. cholerae,* and *V. alginolyticus* that enabled absolute quantification of the results.

The choice of specific targets is a critical element in nucleic acid amplification testing, as it directly affects the specificity of the analytical method. Traditionally, in *Vibrio*, many genes encoding DNA gyrase (*gyrB*), virulence factors (*tlh*, *toxR*, *ctxA*), and outer membrane protein (*ompW*) are employed as specific biomarkers. However, these genes exhibit considerable homology to sequences from genetically related species such as *V. parahaemolyticus*, *V. vulnificus*, *V. cholerae,* and *V. alginolyticus*, indicating that accurate differentiation by conventional gene-based detection methods is difficult. Many researchers have reported that some of these genes are highly conserved or absent in certain environmental settings, potentially leading to misidentification [40,41,42]. Similarly, 16S rRNA gene-based sequencing is another important method widely used for microbial identification and is commonly used to explore genomic diversity and evolutionary relationships based on sequence data in this region [43]. While MALDI-TOF MS detects predominantly ribosomal proteins, its results are compared against those of a standard fingerprint database [44,45]. However, both methods often suffer from cross-reactions because of their phylogenetic proximity. Therefore, it is critically necessary to discover new species-specific genetic markers for the detection of four *Vibrio* species. With the increasing availability of complete whole-genome sequences, it has become feasible to perform comparative genomic analysis for the discovery of unique, novel species-specific gene markers. In our previous study, the species-specific genes *vpa1585* (*V. parahaemolyticus*), *vv08030* (*V. vulnificus*), and *va01740* (*V. alginolyticus*) were screened and verified [27]. Furthermore, a species-specific gene, *vc09280*, encoding a UDP-2,3-diacylglucosamine hydrolase, was first selected in this study for the identification of *V. cholerae*. Following this, the combined use of four target genes can dramatically improve the specificity and efficiency of molecular detection.

As reflected in the National Food Safety Standard (GB 29921-2021, GB 31607-2021) [46,47], maximum limit regulations have been established for common foodborne pathogens, including *V. parahaemolyticus* and *Staphylococcus aureus*. This indicates increased demand for pathogen detection, which should enable not only reliable identification but also accurate quantitative determination. Although qPCR has recently gained attention and recognition due to its real-time observation characteristics, its sensitivity is not always satisfactory. Compared with qPCR, dPCR shows superior capability for the detection of low-abundance targets. Currently, several commercially available dPCR systems, such as QX200™ (Bio-Rad, USA), QuantStudio™ Absolute Q™ (Thermo Fisher Scientific, USA), and QIAcuity (QIAGEN, Germany), are available [48]. To our knowledge, there are no reports on the use of multiplex cdPCR combined with self-screened genes to detect four *Vibrio* species. In this research, 4-plex cdPCR and 4-plex qPCR methods were established. The performance of 4-plex cdPCR was comprehensively optimized by testing combinations of primer/probe and annealing temperatures to balance fluorescence intensity and droplet separation. The assessment results confirmed that the proposed methods achieved 100% accuracy for all the tested strains. The LOD of the 4-plex cdPCR was on the order of 10^2^ CFU/mL for pure culture, indicating a 10-fold improvement over than that of qPCR (10^3^ CFU/mL), which is similar to the results of other published reports [23,49]. However, although multitarget detection has certain technical advantages, its sensitivity is lower than that of single-target detection [21], which might be due to the mutual interference of various targets in the same system. Notably, during the detection process, the conversion relationship between bacterial concentrations and gene copy numbers can be affected by multiple factors, such as cell viability, physiological state, and DNA extraction efficiency. *Vibrio* spp. are the major microbial causative agents of foodborne disease outbreaks, which are caused mainly by the consumption of raw or ready-to-eat aquatic products. Thus, the applicability of our detection methods was evaluated in artificially contaminated samples, further confirming the superiority of the cdPCR technique.

We recognize there are some limitations to this study, particularly that this work is a methodological study conducted in the laboratory. This requires further validation using more natural matrices, alongside systematic assessment of repeatability, reproducibility, and variability. Moreover, the high cost of cdPCR system hinders its dissemination in routine food-testing laboratories. Hence, the selection among different technologies (such as cdPCR, qPCR, and isothermal amplification) is influenced by various factors including the objectives of the study, portability, operation time, and practical considerations such as cost and throughout. Despite our satisfactory results, there is still room for improvement in the LOD of the simultaneous detection of multiple targets. In future studies, appropriate separation and enrichment methods need to be explored according to the actual purposes and demands, and combined with propidium monoazide (PMA) and other nucleic acid dyes to achieve the detection of live bacteria.

## 5. Conclusions

In this study, we successfully developed a novel multiplex cdPCR method for the simultaneous detection of *V. parahaemolyticus*, *V. vulnificus*, *V. cholerae* and *V. alginolyticus* on a single chip. The use of self-screened genes (*vpa1585*, *vv08030*, *vc09280* and *va01740*) has laid an important foundation for accurate detection. Through systematic optimization and evaluation, this method displayed a broad linear range, with a low LOD of 10^2^ CFU/mL for all the target bacteria. Moreover, compared with mainstream technologies (qPCR), the proposed detection system not only enables absolute quantification but also effectively reduces analysis time, making it a reliable solution for the surveillance of pathogenic *Vibrio* species in the aquatic product industry, particularly in situations where multiplex detection is needed.

## Figures and Tables

**Table 1:** Strains used for specificity evaluation.

Strain Category	Strain No.	Detection Results			
		*vpa1585*	*vv08030*	*vc09280*	*va01740*
*V. parahaemolyticus*	ATCC 17802	+	−	−	−
*V. vulnificus*	ATCC 27562	−	+	−	−
*V. cholerae*	CICC 23794	−	−	+	−
*V. alginolyticus*	ATCC 17749	−	−	−	+
*Staphylococcus aureus*	ATCC 25923	−	−	−	−
*Shigella flexneri*	CMCC(B) 51572	−	−	−	−
*Listeria monocytogenes*	ATCC 19115	−	−	−	−
*Escherichia coli*	ATCC 25922	−	−	−	−
*Cronobacter sakazakii*	ATCC 29544	−	−	−	−
*Pseudomonas aeruginosa*	ATCC 9027	−	−	−	−
*Salmonella* Typhimurium	ATCC 14028	−	−	−	−
*V. metschnikovii*	ATCC 700040	−	−	−	−
*V. mimicus*	ATCC 33653	−	−	−	−
*V. fluvialis*	ATCC 33809	−	−	−	−
*V. hollisae*	ATCC 33564	−	−	−	−
*V. harveyi*	ATCC 33842	−	−	−	−
*V. parahaemolyticus*	Vp1–Vp8	+	−	−	−
*V. vulnificus*	Vv1–Vv8	−	+	−	−
*V. cholerae*	Vc1–Vc7, Vc9	−	−	+	−
*V. alginolyticus*	Va1–Va8	−	−	−	+

“+” indicates positive result; “−” indicates negative result.

**Table 2:** Primers and probes used in this study.

Name	Sequence (5′–3′)	Product Size (bp)	References
*vpa1585*-F	ATCTGTTCGTCGTCATTAGCG	186	This study
*vpa1585*-R	CGATGGTTTGCCCTTCCT		
*vpa1585*-P	FAM-TTTGGCTCTATGACTTCCGTTTATCC-BHQ1		
*vv08030*-F	CAACTACTGCGAGTGGTTTCC	151	[27]
*vv08030*-R	CCATGCTTCAGCGGGTCT		
*vv08030*-P	HEX-ACTTGCTTGGCTCACCCGACTC-BHQ1		
*vc09280*-F	TATTTGGACTTCTTCCTTTTGCA	123	This study
*vc09280*-R	CGATGACTTCGCTGGGTGT		
*vc09280*-P	TAMRA-TCCGCAAAATACAGTCAGACATACGAGA-BHQ2		
*va01740*-F	CGCCTTTACTGGTGAGCCT	191	This study
*va01740*-R	GAGCACGAGCAACGATTTCT		
*va01740*-P	CY5-TTCACTTCCAATTCAGAGGCGATAGA-BHQ2		

**Table 3:** Detection of *Vibrio* species in artificially contaminated salmon.

Target Bacteria	Expected Values (CFU/mL)	4-Plex cdPCR		4-Plex qPCR	
		Measured Values (CFU/mL)	RSD (%)	Measured Values (CFU/mL)	RSD (%)
*V. parahaemolyticus*	0	ND	N/A	ND	N/A
	4.36 × 10^1^	ND	N/A	ND	N/A
	4.36 × 10^2^	8.64 × 10^2^	23.82	ND	N/A
	4.36 × 10^3^	3.74 × 10^3^	11.32	2.72 × 10^3^	17.48
	4.36 × 10^4^	2.45 × 10^4^	7.02	3.37 × 10^4^	15.68
	4.36 × 10^5^	3.26 × 10^5^	5.11	3.15 × 10^5^	8.89
	4.36 × 10^6^	3.70 × 10^6^	4.77	3.42 × 10^6^	6.24
*V. vulnificus*	0	ND	N/A	ND	N/A
	2.65 × 10^1^	ND	N/A	ND	N/A
	2.65 × 10^2^	1.21 × 10^2^	16.86	ND	N/A
	2.65 × 10^3^	4.83 × 10^3^	15.43	1.96 × 10^3^	22.61
	2.65 × 10^4^	1.28 × 10^4^	11.85	1.13 × 10^4^	13.10
	2.65 × 10^5^	1.62 × 10^5^	5.12	1.20 × 10^5^	9.41
	2.65 × 10^6^	2.08 × 10^6^	5.85	1.58 × 10^6^	8.23
*V. cholerae*	0	ND	N/A	ND	N/A
	5.46 × 10^1^	ND	N/A	ND	N/A
	5.46 × 10^2^	8.64 × 10^2^	23.82	ND	N/A
	5.46 × 10^3^	6.59 × 10^3^	18.54	3.35 × 10^3^	19.12
	5.46 × 10^4^	4.32 × 10^4^	17.65	2.23 × 10^4^	11.99
	5.46 × 10^5^	2.61 × 10^5^	6.89	3.27 × 10^5^	9.37
	5.46 × 10^6^	4.08 × 10^6^	8.45	3.63 × 10^6^	8.09
*V. alginolyticus*	0	ND	N/A	ND	N/A
	3.24 × 10^1^	ND	N/A	ND	N/A
	3.24 × 10^2^	9.60 × 10^2^	20.94	ND	N/A
	3.24 × 10^3^	2.88 × 10^3^	21.73	1.79 × 10^3^	20.02
	3.24 × 10^4^	1.05 × 10^4^	11.37	1.96 × 10^4^	14.26
	3.24 × 10^5^	1.03 × 10^5^	7.45	2.30 × 10^5^	11.19
	3.24 × 10^6^	2.59 × 10^6^	4.19	2.07 × 10^6^	7.28

ND, Not detected. N/A, Not applicable.

## Data Availability

The original contributions presented in this study are included in the article/Supplementary Materials. Further inquiries can be directed to the corresponding authors.

## Supplementary Materials

The following supporting information can be downloaded at: https://www.mdpi.com/article/10.3390/foods15142547/s1. Table S1. L_9_(3^4^) orthogonal design used for optimization of the probe concentration. Table S2. Detection of *Vibrio* species in artificially contaminated shrimp. Table S3. Detection of *Vibrio* species in artificially contaminated oysters. Table S4. Detection of *Vibrio* species in artificially contaminated large yellow croaker.
