# Supplementary material for: Simultaneous, Quantitative Detection of Four Common Vibrio Species by Microfluidic Chamber-Based Digital PCR in Aquatic Products
Source: Foods. 2026 Jul 19;15(14):2547. doi: 10.3390/foods15142547 (PMC13407433; doi:10.3390/foods15142547)
Supplement: Supplementary file 1 [file foods-15-02547-s001.zip › foods-4401567-supplementary.pdf]

Support Information

Simultaneous, quantitative detection of four common *Vibrio* species by microfluidic chamber-based digital PCR in aquatic products

Haibo Zhou[a,1], Na Wang[b,1], Xinmei Liu[a], Ning Liu[b], Xiaomei Bie[b,*], Jun Yang[a,*]

[a] Key Laboratory of Detection and Traceability Technology of Foodborne Pathogenic Microorganisms, State Administration for Market Regulation, Key Laboratory of Detection and Traceability Technology of Foodborne Pathogenic Bacteria for Jiangsu Province Market Regulation, Nanjing Institute for Food and Drug Control, Nanjing, 211198, China

[b] College of Food Science and Technology, Nanjing Agricultural University, Nanjing 210095, China

E-mail addresses: bxm43@njau.edu.cn (X. Bie), yj711003@sina.com (J. Yang).

Table S1. $L_9(3^4)$ orthogonal design used for optimization of the probe concentration.

**Experiment No.**	**Probe concentration (μM)**			
	vpa1585	***vv08030***	***vc09280***	***va01740***
1	0.05	0.30	0.25	0.50
2	0.05	0.35	0.35	0.55
3	0.05	0.40	0.30	0.60
4	0.10	0.30	0.35	0.60
5	0.10	0.35	0.30	0.50
6	0.10	0.40	0.25	0.55
7	0.15	0.30	0.30	0.55
8	0.15	0.35	0.25	0.60
9	0.15	0.40	0.35	0.50

Table S2. Detection of *Vibrio* species in artificially contaminated shrimp.

Target bacteria	Expected values (CFU/mL)	4-plex cdPCR		4-plex qPCR	
		Measured values (CFU/mL)	RSD (%)	Measured values (CFU/mL)	RSD (%)
V. parahaemolyticus	0	ND	N/A	ND	N/A
	4.36×10^{1}	ND	N/A	ND	N/A
	4.36×10^{2}	1.21×10^{2}	20.94	ND	N/A
	4.36×10^{3}	5.20×10^{3}	13.26	2.72×10^{3}	21.01
	4.36×10^{4}	2.85×10^{4}	10.38	3.54×10^{4}	14.32
	4.36×10^{5}	3.36×10^{5}	9.05	4.09×10^{5}	9.09
	4.36×10^{6}	4.16×10^{6}	5.16	3.83×10^{6}	6.95
V. vulnificus	0	ND	N/A	ND	N/A
	2.65×10^{1}	ND	N/A	ND	N/A
	2.65×10^{2}	1.51×10^{2}	16.86	ND	N/A
	2.65×10^{3}	8.16×10^{3}	18.62	1.90×10^{3}	22.50
	2.65×10^{4}	1.60×10^{4}	11.85	1.06×10^{4}	17.35
	2.65×10^{5}	2.02×10^{5}	10.87	1.36×10^{5}	10.50
	2.65×10^{6}	2.59×10^{6}	6.10	1.54×10^{6}	3.96
V. cholerae	0	ND	N/A	ND	N/A
	5.46×10^{1}	ND	N/A	ND	N/A
	5.46×10^{2}	1.21×10^{2}	20.94	ND	N/A
	5.46×10^{3}	1.71×10^{3}	15.95	3.08×10^{3}	20.13
	5.46×10^{4}	3.68×10^{4}	13.45	2.03×10^{4}	18.75
	5.46×10^{5}	4.47×10^{5}	9.19	3.44×10^{5}	14.82
	5.46×10^{6}	5.19×10^{6}	7.04	5.13×10^{6}	7.78
V. alginolyticus	0	ND	N/A	ND	N/A
	3.24×10^{1}	ND	N/A	ND	N/A
	3.24×10^{2}	1.06×10^{2}	23.82	ND	N/A
	3.24×10^{3}	5.20×10^{3}	13.26	1.52×10^{3}	16.43
	3.24×10^{4}	2.08×10^{4}	9.99	1.99×10^{4}	14.49
	3.24×10^{5}	1.42×10^{5}	14.11	2.54×10^{5}	11.99
	3.24×10^{6}	2.93×10^{6}	7.61	2.50×10^{6}	8.73

ND, Not detected. N/A, Not applicable.

Table S3. Detection of *Vibrio* species in artificially contaminated oysters.

Target bacteria	Expected values (CFU/mL)	4-plex cdPCR		4-plex qPCR	
		Measured values (CFU/mL)	RSD (%)	Measured values (CFU/mL)	RSD (%)
V. parahaemolyticus	0	ND	N/A	ND	N/A
	4.36×10^{1}	ND	N/A	ND	N/A
	4.36×10^{2}	1.08×10^{2}	20.94	ND	N/A
	4.36×10^{3}	1.47×10^{3}	17.07	1.44×10^{3}	22.53
	4.36×10^{4}	1.48×10^{4}	11.32	1.28×10^{4}	20.92
	4.36×10^{5}	1.36×10^{5}	7.63	1.24×10^{5}	11.50
	4.36×10^{6}	2.53×10^{6}	4.43	1.52×10^{6}	8.27
V. vulnificus	0	ND	N/A	ND	N/A
	2.65×10^{1}	ND	N/A	ND	N/A
	2.65×10^{2}	9.72×10^{2}	23.82	ND	N/A
	2.65×10^{3}	2.59×10^{3}	24.74	1.68×10^{3}	21.14
	2.65×10^{4}	1.83×10^{4}	14.76	1.30×10^{4}	15.71
	2.65×10^{5}	1.62×10^{5}	5.60	1.76×10^{5}	8.87
	2.65×10^{6}	1.62×10^{6}	2.57	1.34×10^{6}	7.63
V. cholerae	0	ND	N/A	ND	N/A
	5.46×10^{1}	ND	N/A	ND	N/A
	5.46×10^{2}	1.36×10^{2}	16.86	ND	N/A
	5.46×10^{3}	5.18×10^{3}	13.94	1.62×10^{3}	15.96
	5.46×10^{4}	3.29×10^{4}	8.57	2.64×10^{4}	10.93
	5.46×10^{5}	3.49×10^{5}	7.59	2.94×10^{5}	9.72
	5.46×10^{6}	4.66×10^{6}	3.10	4.10×10^{6}	6.19
V. alginolyticus	0	ND	N/A	ND	N/A
	3.24×10^{1}	ND	N/A	ND	N/A
	3.24×10^{2}	1.08×10^{2}	20.94	ND	N/A
	3.24×10^{3}	4.21×10^{3}	19.02	2.52×10^{3}	21.32
	3.24×10^{4}	1.73×10^{4}	11.30	1.56×10^{4}	13.11
	3.24×10^{5}	1.56×10^{5}	7.27	1.52×10^{5}	7.47
	3.24×10^{6}	2.55×10^{6}	3.80	1.87×10^{6}	4.87

ND, Not detected. N/A, Not applicable.

Table S4. Detection of *Vibrio* species in artificially contaminated large yellow croaker.

Target bacteria	Expected values (CFU/mL)	4-plex cdPCR		4-plex qPCR	
		Measured values (CFU/mL)	RSD (%)	Measured values (CFU/mL)	RSD (%)
V. parahaemolyticus	0	ND	N/A	ND	N/A
	4.36×10^{1}	ND	N/A	ND	N/A
	4.36×10^{2}	1.29×10^{2}	16.86	ND	N/A
	4.36×10^{3}	1.36×10^{3}	15.20	1.14×10^{3}	16.97
	4.36×10^{4}	1.21×10^{4}	11.06	1.08×10^{4}	12.66
	4.36×10^{5}	1.49×10^{5}	9.58	1.21×10^{5}	12.87
	4.36×10^{6}	2.21×10^{6}	4.48	1.96×10^{6}	6.92
V. vulnificus	0	ND	N/A	ND	N/A
	2.65×10^{1}	ND	N/A	ND	N/A
	2.65×10^{2}	1.02×10^{2}	20.94	ND	N/A
	2.65×10^{3}	1.53×10^{3}	21.74	1.05×10^{3}	21.93
	2.65×10^{4}	1.72×10^{4}	11.22	1.33×10^{4}	13.19
	2.65×10^{5}	1.01×10^{5}	11.61	1.06×10^{5}	10.99
	2.65×10^{6}	1.48×10^{6}	5.74	1.26×10^{6}	6.59
V. cholerae	0	ND	N/A	ND	N/A
	5.46×10^{1}	ND	N/A	ND	N/A
	5.46×10^{2}	9.18×10^{2}	23.82	ND	N/A
	5.46×10^{3}	3.57×10^{3}	19.63	3.14×10^{3}	22.09
	5.46×10^{4}	2.99×10^{4}	15.10	2.29×10^{4}	16.97
	5.46×10^{5}	3.19×10^{5}	7.98	2.81×10^{5}	10.12
	5.46×10^{6}	4.10×10^{6}	4.45	3.84×10^{6}	5.17
V. alginolyticus	0	ND	N/A	ND	N/A
	3.24×10^{1}	ND	N/A	ND	N/A
	3.24×10^{2}	1.02×10^{2}	20.94	ND	N/A
	3.24×10^{3}	3.19×10^{3}	21.36	2.31×10^{3}	22.30
	3.24×10^{4}	1.47×10^{4}	10.51	1.25×10^{4}	18.52
	3.24×10^{5}	1.38×10^{5}	9.42	1.03×10^{5}	11.14
	3.24×10^{6}	2.14×10^{6}	4.84	1.84×10^{6}	5.02

ND, Not detected. N/A, Not applicable.
